# Nanotechnology-driven advances in intranasal vaccine delivery systems against infectious diseases

**DOI:** 10.3389/fimmu.2025.1573037

**Published:** 2025-05-09

**Authors:** Zhihan Zhang, Yumeng Yang, Liwen Huang, Lei Yuan, Sijian Huang, Zihang Zeng, Yuan Cao, Xianghong Wei, Xiaomei Wang, Mingsong Shi, Maohua Zhong

**Affiliations:** ^1^ Institute of Infection, Immunology and Tumor Microenvironment, Hubei Province Key Laboratory of Occupational Hazard Identification and Control, Medical College, Wuhan University of Science and Technology, Wuhan, Hubei, China; ^2^ Analytical & Testing Center, Wuhan University of Science and Technology, Wuhan, Hubei, China; ^3^ NHC Key Laboratory of Nuclear Technology Medical Transformation, Mianyang Central Hospital, School of Medicine, University of Electronic Science and Technology of China, Mianyang, Sichuan, China

**Keywords:** infectious disease, vaccine, mucosal vaccination, intranasal immunization, delivery system

## Abstract

Outbreaks of emerging and re-emerging infectious diseases have consistently threatened human health. Since vaccinations are a powerful tool for preventing infectious illnesses, developing new vaccines is essential. Compared to traditional injectable vaccines, mucosal vaccines have the potential to offer more effective immune protection at mucosal sites. Mucosal immunization strategies include sublingual, oral, intranasal, genital, and rectal routes, in which intranasal immunization being the most efficient and applicable method for mucosal vaccine delivery. Nevertheless, low antigen availability and weak immunogenicity making it challenging to elicit a potent immune response when administered intranasally, necessitating the incorporation of immune delivery systems. However, there is a notable absence of reviews that summarize the intranasal vaccine delivery system against infectious disease. Therefore, this review summarizes the recent advances in intranasal delivery systems, classified by physical and chemical properties, and proposes potential improvement strategies for clinical translation. This review elucidates the potential and current status of intranasal delivery systems, while also serving as a reference point for the future development of intranasal vaccines.

## 1 Introduction

Infectious diseases including influenza, acquired immune deficiency syndrome (AIDS), measles, and coronavirus disease 2019 (COVID-19) pose persistent threats to global public health, necessitating urgent advancements in prophylactic strategies (1). While medical advancements over the past two decades have reduced mortality through improved sanitation and healthcare infrastructure (2), the emergence of novel pathogens and antimicrobial resistance underscores the need for innovative immunization approaches. As the cornerstone of infectious disease prevention, vaccines function by priming innate immunity to activate antigen-specific adaptive responses through antibody production and T-cell mediation—a dual mechanism that curbs pathogen transmission while protecting vulnerable populations (3).

Current vaccine delivery modalities can be broadly categorized into systemic injection and mucosal immunization. In contrast to traditional injectable vaccines that primarily elicit systemic immunity, mucosal vaccines offer dual protective advantages: 1) induction of both systemic and mucosal immune responses, 2) generation of secretory IgA and tissue-resident memory T cells at portal-of-entry mucosal sites, and 3) needle-free administration that enhances safety and compliance (4, 5). Given that >90% of pathogens invade through mucosal surfaces, mucosal immunization serves as the primary immunological barrier against infection establishment (6).

Among mucosal delivery routes—including oral, buccal, sublingual, intranasal, and genital approaches—intranasal immunization stands out as a particularly promising candidate. This preference stems from the nasal cavity’s unique immunological architecture: 1) dense networks of microfold (M) cells and antigen-presenting cells (APCs) within nasal-associated lymphoid tissue (NALT), 2) vascularized subepithelial layers facilitating rapid systemic absorption, and 3) interconnected mucosal immunity enabling cross-protection at distal sites such as pulmonary and intestinal mucosa (7). Notably, currently approved intranasal vaccines predominantly target influenza, including FluMist (US) (8) and Nasovac-S (India) (9). The COVID-19 pandemic further accelerated clinical development of intranasal SARS-CoV-2 vaccines (10), with emergency approvals granted for formulations acting as primary immunogens or heterologous boosters to enhance mucosal immunity and tissue-resident memory T cells (11) (**Table 1**).

**Table 1:** Currently approved intranasal vaccines.

Disease	Vaccine	Type	Country	Links
influenza	FluMist	Live attenuated vaccine	MedImmune, LLC	https://www.fda.gov/media/180697/download?attachment
	FluMist Quadrivalent	Live attenuated vaccine	MedImmune, LLC	https://www.fda.gov/media/160349/download?attachment
	Nasovac-S4	Live attenuated vaccine	Serum Institute of India	https://www.seruminstitute.com/product_ind_NASOVAC-S4.php
COVID-19	BBV154	adenovirus vector vaccine	Bharat Biotech	https://www.bharatbiotech.com/intranasal-vaccine.html

The data is derived from FDA, Serum Institute of India, Bharat Biotech provided in the websites.

The clinical advantages of intranasal vaccines are multifaceted: (i) Non-invasive administration: Intranasal vaccines are an excellent non-invasive vaccination method (7, 12). (ii) Induction of mucosal immunity: In addition to systemic immune response, intranasal vaccines can act on the extensive respiratory mucosa, eliciting mucosal immunity and providing more comprehensive and effective protection for the human body (12). (iii) Improved pediatric compliance: compared to the pain caused by intramuscular injections, nasal drops offer a more comfortable vaccination experience for children and are also suitable for other individuals with needle or pain phobias (13, 14). (iv) Operational and economic benefits: intranasal vaccines offer greater practicality in terms of cost and ease of administration (15). While transient adverse effects (e.g., rhinorrhea, low-grade fever) occur in some recipients, these typically resolve without intervention (16).

**Figure 1:**
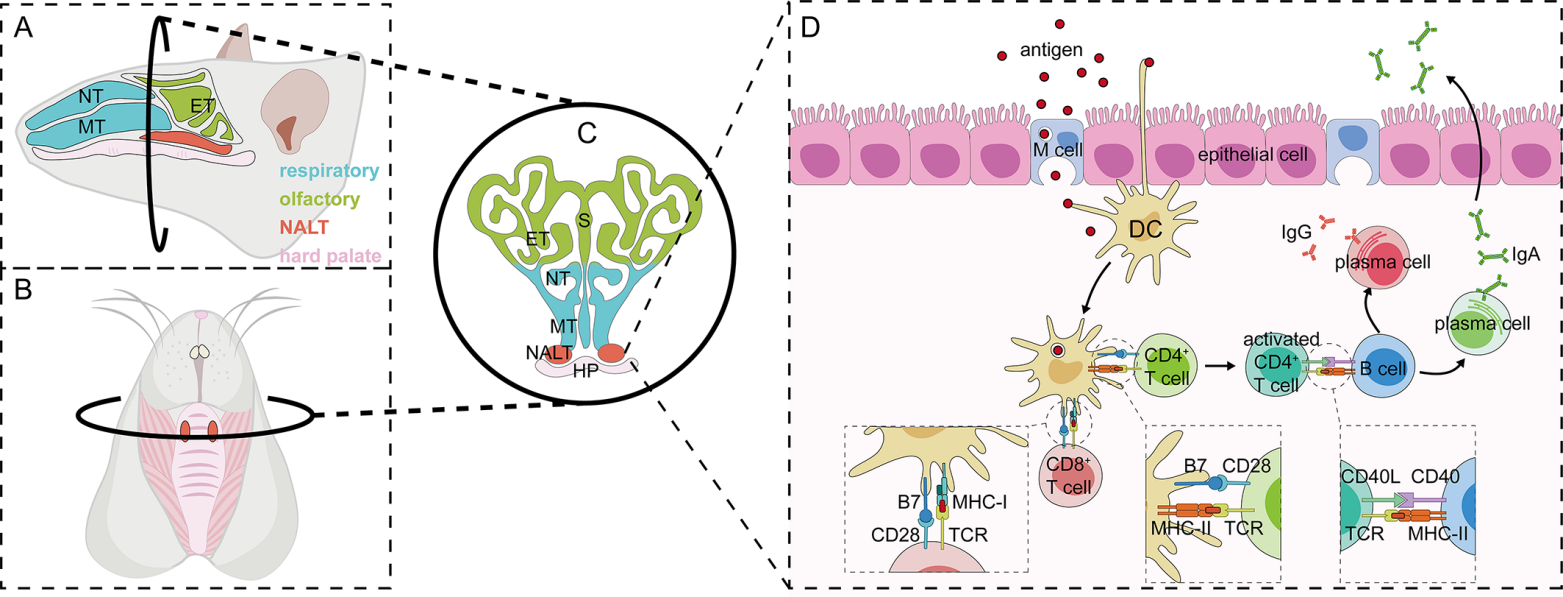
Anatomy of and immune induction in nasal-associated lymphoid tissues in rodents. **(A-C)** Anatomical location of NALT: The sagittal anatomy of mouse nasal tissues can be divided into two distinct regions—the respiratory and olfactory parts—based on the structure and function of the mucosa **(A)**. A specialized structure in the nasal tissue, the nasal mucosa-associated lymphoid tissue (NALT), consists of two parallel bell-shaped formations located above the hard palate **(B, C)**. Immune response in NALT: The immune response in NALT progresses through three main stages: antigen uptake and presentation, immune cell activation, and the subsequent immune response. Antigens entering the nasal cavity are first internalized by microfold cells (M cells), which then transport them into NALT, where dendritic cells further process and present the antigens. In addition, some dendritic cells are capable of crossing the epithelial barrier by extending their dendrites, allowing them to directly capture antigens. Antigen-loaded dendritic cells activate both CD4^+^ and CD8^+^ T cells upon antigen presentation. Activated CD4^+^ T cells further stimulate B cells via CD40L/CD40 interaction, promoting their differentiation into plasma cells. These fully differentiated plasma cells subsequently initiate an immune response at the effector site through the production of IgA and IgG antibodies **(D)**. NT, nasoturbinate; MT, maxillary turbinate; ET, ethmoid turbinate; S, septum; HP, hard palate.

The nasal cavity is a complex anatomical structure with superior, inferior, medial, and lateral walls. The two nasal passages have a combined surface area of about 160 cm^2^ (96 m^2^ if nasal epithelial microvilli are included), and a combined volume of about 15 mL. The nasal cavity can be divided into three main regions: the nasal vestibule, the respiratory tract, and the olfactory region. The nasal septum, which separates the two nasal passages, forms the medial wall of the nasal cavity. The superior, middle, and inferior turbinates, which delineate the upper, middle, and lower nasal passages respectively, constitute the lateral wall of the nasal cavity (17). The human Waldeyer’s ring, comprising the adenoid, lingual tonsil, two palatine tonsils, and two tubal tonsils, is believed to be analogous to the NALT (nasal-associated lymphoid tissue) observed in rodents (8). The NALT can be defined as an organized mucosal-associated lymphoid tissues within the nasal mucosa, consisting of lymphoid tissue, B cells, T cells, antigen-presenting cells (APCs) and microfold (M) cells. M cells are specifically for antigen uptake (9). NALT plays a critical role in antigen recognition and immune activation following intranasal immunization, serving as a key site for immune induction within the nasal mucosa (**Figure 1**).

Low antigen availability and weak immunogenicity, combined with antigen clearance by nasal cilia, the mucous layer barrier, and tight junctions between epithelial cells, significantly reduce vaccine uptake. This hinders the elicitation of a robust immune response, thereby posing challenges for nasal administration (18). The nasal drug delivery system (NDDS) is a needle-free, non-invasive, and efficient method for delivering drugs, vaccines, and other therapeutic agents into the human body via the nasal route which include nanoparticles (**Tables 2**, **3**), nanoemulsions, and some microbial preparations (**Table 4**) (12, 19). Among them, nanoparticles are the most widely used, which include polysaccharide-based particles, poly lactide-co-glycolide (PLGA), gold nanoparticles, nanogels, lipopeptides, liposomes and others (19, 20). Through boosting antigen penetration, shielding the antigen from degradation, facilitating sustained antigen release, improving nasal retention time, and recruiting/activating APCs, the nasal delivery system can significantly increase the antigen availability and immunogenicity (18). The NDDS offers the advantage of boosting vaccination efficiency against pathogens through the induction of both systemic and mucosal immune responses at respiratory and distal mucosal sites (21).

**Table 2:** Preclinical studies of intranasal vaccines using polysaccharides as an intranasal delivery system.

Type of particle	Species	Antigen	Immunization parameters	Key outcome	Ref.
chitosan	BALB/c mice	SARS-CoV-2 S-RBD	CS & O-HTCC/5μg of S-RBD/immunotherapy in three doses with 14-day intervals	↑of the humoral, mucosal and cellular immune responses. CS/S-RBD/O-HTCC with two antigens could induced neutralizing antibodies against both the ancestral and mutated S-RBD	(25)
	BALB/c mice	recombinant H1N1 hemagglutinin protein	C-O-NP/20 μL(containing 1.5μg HA)/immunotherapy in 2 doses on days 0 and 14	↑of the cellular immune responses Stimulated proximal and distal mucosal immune responses through antigen-specific sIgA in both saliva and vaginal lavage fluid	(30)
	C57BL/6 mice	HBsAg	Chi-C48/80 NPs/15μL of vaccine formulation (7.5µL per nostril)/immunotherapy in 3 doses on Days 0, 7, and 21	Induced humoral and mucosal antibodies against HBV (anti-HBsAg IgA, specific IgG)	(32)
	BALB/c mice	HBsAg	GC NPs/immunotherapy in 2 doses on Days 0 and 21	Elicited stronger humoral and mucosal immune response and lower rate of clearance from the nasal cavity compared to chitosan NPs after nasal administration	(31)
Starch	Balb/c mice	Human serum albumin (HSA)	HSA containing TS-PDMS-grafted MP/10 ul per dose/intranasally vaccinated on days 0, 7 and 14	Elevated anti-HSA serum IgG levels	(36)
Pullulan	Balb/c mice	pneumococcal surface protein A (PspA)	cationic cholesteryl group-bearing pullulan nanogel with PspA/Once weekly for 3 consecutive weeks	Elevated anti-PspA serum IgG and mucosal PspA-specific sIgA levels and enhanced bacterial clearance from BALF and the lung	(42)
	cynomolgus macaques	PspA	cationic cholesteryl group-bearing pullulan nanogel with PspA/25 ug per dose/nasally immunized five times at 2-week intervals	PspA-specific bronchoalveolar lavage fluid IgG and nasal wash IgA responses exhibited higher levels	(41)
Maltodextrin	CBA/J (H-2k) mice	Toxoplasma gondii antigen extract (TE)	Maltodextrin (MD)-loaded TE/three doses intra-nasally at 2-week intervals of DGNP, free TE, CT, CT/TE and DGNP/TE (6μL/nostril)	Higher levels of total IgG, the mixed IgG1/IgG2a response and a mixed Th1/Th17 response Protective immune response: higher reduction rate of TE cysts, reducing parasite growth and protecting mice against long-term infection	(50)
	BALB/c and DBA/2J mice	split Udorn virus antigens	Maltodextrin (MD)-loaded split Udorn virus antigens/three times, 10 days apart of split Udorn only, split Udorn/NPL, split Udorn/CTA1-DD/NPL(5μl/nostril)	Increased intracellular antigen delivery and specific humoral immune response Decreased viral titers Inhibited viral transmission	(51)
Alginate	pigs	Bee venom	chitosan/alginate nanoparticles double encapsulated 1mg/2mg of BV and administered it one week before PRRSV challenge and 2 weeks after the challenge	Showed significant increases in the populations of Th cells and PRRSV-specific IgG Ab levels. Viral loads in serum and tissues with PRRSV challenge decreased.	(54)

SARS-CoV-2, Severe Acute Respiratory Syndrome Coronavirus 2; S-RBD, spike receptor binding domain; CS, curdlan sulfate; O-HTCC, O-(2-hydroxyl) propyl-3-trimethyl ammonium chitosan chloride; C-O-NP, curdlan-chitosan conjugate nanoparticle; HA, hyaluronic acid; HBsAg, hepatitis B surface antigen; Chi-C48/80, compound 48/80 loaded chitosan; HBV, hepatitis B virus; IgA, immunoglobulin A; IgG, immunoglobulin G; GC, glycol chitosan; HAS, human serum albumin; TS-PDMS-grafted MP, 3-(triethoxysilyl)-propyl-terminated polydimethylsiloxane-grafted microparticles; PspA, pneumococcal surface protein A; BALF, bronchoalveolar lavage fluid; TE, total extract of Toxoplasma gondii antigens; MD, Maltodextrin; DGNP, Dendritic Gold Nanoparticles; CT, cholera toxin; CTA1-DD, cholera toxin A1 subunit -dimer of the D-fragment of protein A from Staphylococcus aureus bacteria; NPL, nanoparticles lipid; BCV, Bee venom; PRRSV, porcine reproductive and respiratory syndrome virus.

**Table 3:** Preclinical studies of other nanoparticles used in intranasal delivery system.

Type of particle	Species	Antigen	Immunization parameters	Key outcome	Ref.
PLGA	mouse (J774) and human (THP-1) macrophage cell lines	The antigenic peptide epitope of the ESAT-6 protein of M. tuberculosis	PVP coated PLGA nanoparticle	Augmented the macrophages with much less quantity of the antigenic peptide	(63)
AuNPs	transgenic C57BL/6J-DR and BALB/c mice	SARS-CoV-2	SARS-CoV-2-spike DNA vaccine transported on a modified gold-chitosan nanocarrier/20 micrograms of DNA	AuNP-chitosan- SARS-CoV-2-spike DNA vaccines effectively induced S-antigen specific IgG, IgA, and IgM responses in immunocompetent mice	(69)
	C57BL/6 mice	Bm	nano-glycoconjugate vaccine/10 μg of protein and 10 μg of LPS coupled to AuNPs and 20 μg of CpG	Enhanced specific IgG titer and protection-related Th1-biased immune responses	(70)
Nano gel	C57BL/6 mice	–	a gel system composed of a derivative of glutamine amide and benzaldehyde/given EVs hydrogel/solution intranasally, 2 μL each time	Reduced the levels of pro-inflammatory Ly6C (high) monocytes/macrophages and neutrophils	(76)
Lipopeptides	Swiss outbred mice	GAS epitope peptide	Lipopeptides-loaded GAS epitope peptide (LP-88/30-J14, LP-88/30, LP-J14)/first immunization and followed by similar booster doses on days 21 and 41 post primary immunization	Smaller lipopeptides were taken up more readily by APCs Significantly higher serum IgG antibody responses More efficient APC uptake, promoting the maturation of APCs	(79)
Liposomes	mice	H3N2, H1N1	A self-assembling influenza virus vaccine platform that seamlessly converts soluble antigens into nanoparticles	Induction of mucosal antibody responses and production of large amounts of IgA and IgG in respiratory tissues	(86)

PLGA, poly(lactide-co-glycolide); ESAT, Early Secretory Antigenic Target; PVP, poly (4-vinylpyridine); AuNPs, Gold nanoparticles; SARS-CoV-2, Severe Acute Respiratory Syndrome Coronavirus 2; IgA, immunoglobulin A; IgM, immunoglobulin M; IgG, immunoglobulin G; Bm, Burkholderia mallei; LPS, lipopolysaccharide; Th1, type-1 helper T cell; GAS, Group A Streptococcus; LP, Lipopeptides; APCs, antigen presenting cells.

**Table 4:** Preclinical studies for intranasal vaccination with other delivery systems.

Type of particle	Species	Antigen	Immunization parameters	Key outcome	Ref
nanoemulsion	BALB/c mice	Helicobacter pylori HpaA epitope peptide (P22)	NE-loaded HpaA epitope peptide P22/four times at 1-week of the NE, NE-P22, NE-P22 with CpG, P22 with CpG 1826, P22 only, NE with CpG or CpG only(10μL/nostril)	Enhancing the protective efficiency of the epitope vaccine against helicobacter pylori Promoting the maturation of BMDC Slowing the release of P22 *in vitro*, significantly prolonging nasal residence time *in vivo* Effectively enhancing P22 uptake by CD11^+^ DCs in nasal mucosal tissues	(104)
	C57BL/6 mice	Recombinant SARS-CoV-2 spike protein S1 subunit	NE/IVT DI-loaded SARS-CoV-2 spike protein/three times at 4-week intervals of S1 only NE/S1, NE/IVT DI/S1(6μL/nostril)	Higher serum antigen-specific IgG, IgA titers Higher virus-neutralizing antibody titers	(105)
Bacillus subtilis spores	Balb/c mice	Recombinant protein of P. falciparum CSP (rPfCSP)	rPfCSP coupled to B. subtilis spores/10ug per dose/intranasally vaccinated on days 0, 14 and 21	Elevated serum IgG levels, especially IgG2b levels, and induced a balanced Th1/Th2 immune response	(116)
	C57BL/6 mice	C fragment of the tetanus toxin (TTFC)	Spore-adsorbed TTFC/2.0×10^9^ spore-adsorbed with 2.0 µg of TTFC in a volume of 20 µl of 50 mM Sodium Citrate buffer/vaccinated by the intranasal route on day 0 and received a booster on days 14 and 28	Increased sIgA production, accelerated the production of serum IgG	(119)

HpaA, Helicobacter pylori adhesin A; NE, nanoemulsion; SARS-CoV-2, Severe Acute Respiratory Syndrome Coronavirus 2; IVT DI, an RNA agonist of RIG-I; IgA, immunoglobulin A; IgG, immunoglobulin G; rPfCSP, recombinant P. falciparum circumsporozoite surface protein; Th1, type-1 helper T cell; Th2, type-2 helper T cell; TTFC, C fragment of the tetanus toxin; BMDC, Bone Marrow-Derived Dendritic Cells.

Currently, the majority of clinical research on intranasal vaccines focuses on respiratory diseases such as influenza, RSV (Respiratory Syncytial Virus), COVID-19, and whooping cough (**Table 5**). Among these, some vaccines incorporate delivery systems such as nanoemulsions, liposomes, VLPs (Virus-Like Particles), and chitosan to enhance the efficacy of antigens. In these studies, influenza vaccines are the most extensively researched and widely applied intranasal vaccines. The primary type is the live attenuated vaccine, which holds significant research value and market potential, and has also spurred the development of other live attenuated vaccines.

**Table 5:** The current progress in clinical studies of intranasal vaccines.

Disease	Interventions	Delivery system/ adjuvant	ClinicalTrials. gov Identifier	Phase	Sponsor	Status
Norovirus	Norwalk VLP Vaccine	VLP adjuvanted with MPL and chitosan	NCT00806962	1	LigoCyte Pharmaceuticals, Inc.	Completed
HIV	Vacc-4x	Endocine	NCT01473810	1/2	Oslo University Hospital	Completed
	Ad4-mgag Ad4-EnvC150 gp 120 Protein Boost		NCT01989533	1	National Institute of Allergy and Infectious Diseases (NIAID)	Completed
influenza	Influnza vaccination	Liposomes	NCT00197301	1/2	Hadassah Medical Organization	Completed
	Sing2016 M2SR H3N2 influenza vaccine		NCT04785794	1	FluGen Inc	Completed
	BW-1014	Nanoemulsion	NCT05397119	1	BlueWillow Biologics	Completed
	hemagglutinin (HA)	AD07010LTh(αK)	NCT03784885	2	Advagene Biopharma Co. Ltd.	Completed
	A/California/07/09 live monovalent H1N1 vaccine		NCT01023776	4	University of Rochester	Completed
Whooping Cough	Vaccine GamLPV		NCT04036526	1/2	Gamaleya Research Institute of Epidemiology and Microbiology, Health Ministry of the Russian Federation	Unknown status
	BPZE1		NCT03541499	2	National Institute of Allergy and Infectious Diseases (NIAID)	Completed
Covid19	MV-014-212		NCT04798001	1	Meissa Vaccines, Inc.	Unknown status *
	CVXGA1		NCT04954287	1	CyanVac LLC	Completed
RSV	MV-012-968		NCT04444284	1	Meissa Vaccines, Inc.	Completed
	RSVt Vaccine		NCT05687279	1/2	Sanofi Pasteur, a Sanofi Company	Completed
Tuberculosis	TB/Flu-05E		NCT05945498	1	Tatyana Zubkova	Completed
	TB/Flu-05E		NCT06873282	2	Research Institute of Influenza, Russia	Completed
Anthrax	BW-1010	Nanoemulsion	NCT04148118	1	BlueWillow Biologics	Completed
Asthma in Children	LAIV IIV		NCT03600428	4	Vanderbilt University Medical Center	Completed

VLP, Virus-like particle; HIV, Human immunodeficiency virus; Covid-19, Coronavirus disease 2019; RSV, Respiratory syncytial virus; LAIV, Live attenuated influenza vaccine; IIV, Inactivated influenza vaccine; The data us derived from https://clinicaltrials.gov/ provided in the websites.

In this review, we focus on NDDS as advanced carriers for optimizing antigen transport and mucosal retention. While the primary emphasis lies in elucidating mechanistic strategies for targeted delivery, we also examine the combination role of adjuvants. To avoid ambiguity, adjuvants (e.g., TLR agonists, cyclic dinucleotides) is restricted to the components enhancing immune responses although certain adjuvants (e.g., particulate carriers like alum or lipid-based nanoparticles) inherently possess dual functionalities as both immunostimulants and delivery vehicles.

## 2 Nanoparticles types used in nasal vaccines

### 2.1 Polysaccharide: biocompatible and biodegradable polymers for delivery systems

**Figure 2:**
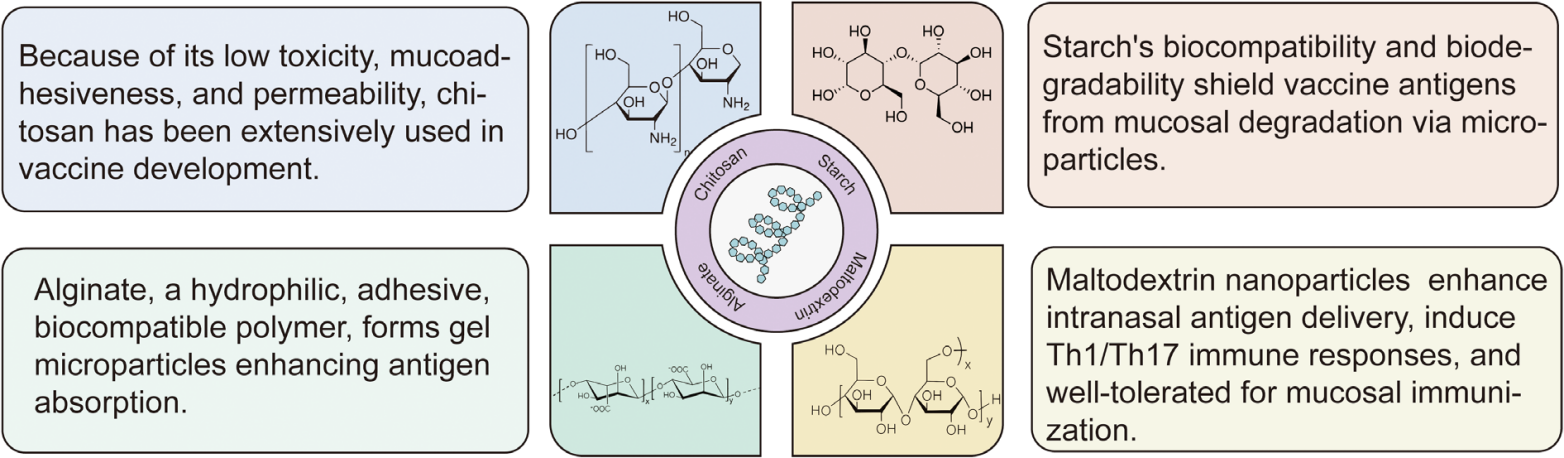
Structural and functional features of polysaccharide-based intranasal delivery systems. The most promising polysaccharide platforms include chitosan, starch, maltodextrin, alginate and others. Low-toxicity and mucoadhesive chitosan improves antigen transport across mucosal barriers; biodegradable starch microparticles protect antigens from enzymatic breakdown; hydrophilic gel microparticles, alginate, aid in the absorption and retention of mucosal antigens; maltodextrin nanoparticles improve intranasal delivery effectiveness, and they induce Th1/Th17 responses with high tolerability.

Polysaccharides represent an exciting class of biomaterials for intranasal vaccine delivery, offering three critical advantages: excellent biocompatibility, predictable biodegradability, and potent immunomodulatory properties (9, 22). The most promising polysaccharide platforms include chitosan, starch, maltodextrin, alginate, etc (**Figure 2**). These innovative materials effectively solve three major challenges in intranasal vaccination: overcoming mucociliary clearance, enhancing epithelial barrier penetration, and providing sustained antigen release (18), all while maintaining superior safety and stability profiles (8). Ongoing research continues to unlock their full potential for developing next-generation mucosal vaccines.

#### 2.1.1 Chitosan

Chitosan is a naturally occurring, biocompatible linear polysaccharide composed of repeating units of d-glucosamine and N-acetyl-d-glucosamine. It is the product of N-deacetylation of chitin, which is derived from the exoskeletons of crustaceans and insects. Chitosan is a safe and biocompatible cationic polymer that can be degraded by a group of chitinases found in many organisms, including humans (23). In addition to its safety and biocompatibility, chitosan has been extensively researched for its low toxicity, mucoadhesiveness, immunogenicity, and permeability. The U.S. Food and Drug Administration (FDA) has approved the use of chitosan in foods and pharmaceuticals (24).

Nanoparticles (NPs) based on chitosan or chitosan derivatives, ranging in size from 1 to 1000 nanometers have been utilized as promising immune delivery systems, delivering vaccine antigens through mucosal pathways such as the nasal routes. Chitosan nanoparticulate systems are particularly successful as vaccine delivery vehicles due to their positive charge, which can improve antigen absorption and boost cellular uptake. Experiments have revealed that positively charged chitosan nanoparticles tend to adhere to negatively charged mucoproteins, reducing nasal evacuation of the nanoparticles. Additionally, Chitosan and its derivatives have the ability to open tight junctions between epithelial cells, elicit immunological responses, and promote IFN-I production, making them potentially effective immune delivery systems (25–27).

In recent years, vaccines based on chitosan or its derivatives as intranasal immune delivery systems have been extensively studied and have been applied in the development of vaccines for infectious disease. For instance, a lentogenic live-virus vaccine (strain LaSota) against newcastle disease virus encapsulated chitosan nanoparticles can elicit robust mucosal immune responses and show a high level of safety in chickens (28).

Although chitosan has significant potential for application, it is sensitive to pH: chitosan dissolves readily at lower pH levels but is less soluble at higher pH levels. Since many proteins are unstable at low pH levels, chitosan’s pH sensitivity may limit its use in antigen delivery (29). Therefore, chitosan is typically modified, such as by forming nanoparticles or allowing free amino and carboxyl functional groups in chitosan through various chemical reactions, including carboxylation, esterification, hydroxylation, and acetylation, to make chitosan derivatives (24).

Among different chitosan derivatives, quaternization, particularly N-trimethyl chitosan (N-TMC), has received the greatest interest. This is owing to its persistent cationic charge and wide pH solubility range. Even at neutral pH, it exhibits strong mucoadhesive and permeation-enhancing properties (26). Chen et al. developed a nanoparticle delivery system, CS/O-HTCC, using the chitosan derivative O-(2-hydroxy) propyl-3-trimethyl ammonium chitosan chloride (O-HTCC) and the β-glucan derivative, curdlan sulfate (CS). They created a nasal mucosal protein subunit vaccine, CS/S-RBD/O-HTCC, using the SARS-CoV-2 spike receptor binding domain (S-RBD) as the antigen, and confirming its safety *in vitro* and *in vivo*. When CS/O-HTCC is loaded with S-RBD mixed antigens (ancestral and omicron), it provoked high levels of ancestral S-RBD specific IgG in serum, sIgA from nasal lavage fluid and antigen-specific IFN-γ from splenic lymphocytes, suggesting that CS/S-RBD/O-HTCC could induce robust humoral, mucosal and cellular immunity. For the neutralizing assay, this vaccination could induce neutralizing antibodies against both ancestral and omicron pseudoviruses than those in the groups using single antigens. This delivery vehicle might be utilized to rapidly prepare vaccines against the continuously mutating SARS-CoV-2 virus (25). In another study, Chen et al. discovered that nanoparticles formed by coupling sulfated lentinan and O-HTCC, as a nasal H1N1 subunit vaccine delivery carrier can stimulate mice macrophage phagocytosis, promote dendritic cell maturation and activation, enhance the production of antigen-specific sIgA in saliva and vaginal lavage fluid and IgG in serum, indicating promising applications (30).

What’s more, numerous recent studies have explored the modification of chitosan nanoparticles with other substances, such as ethylene glycol. Dilip Pawar et al. were the first to synthesize and evaluate glycol chitosan (GC) nanoparticles. In the *in vitro* Calu-3 cell line model, they found that chitosan and GC exhibited minimal toxicity. In comparison to bare chitosan nanoparticles, intranasal treatment of GC nanoparticles loaded with hepatitis B surface antigen (HBsAg) in mice demonstrated better mucoadhesion, which resulted in a substantially lower rate of clearance as compare to chitosan NPs (31).

In addition to surface modification of chitosan to enhance the immune response elicited by vaccines, studies have also explored the adjuvant combination to develop more effective vaccine formulations. Dulce Bento developed a co-adjuvant delivery system for intranasal administration of the hepatitis B virus vaccine, combining the mast cell activator C40/80 with chitosan nanoparticles. The delivery system was shown to have promising features for vaccine delivery, such as the ability to adsorb high amounts of antigen, internalization by APCs, and stability after lyophilization, which can be important during the development of designing a cold chain-free vaccine. C48/80 loaded chitosan nanoparticles NPs have the ability to enhance the immune response to HBsAg such as inducing mucosal anti-HBsAg IgA and specific IgG titers in the vaccinated mice compared to free HBsAg and chitosan-poly epsilon caprolactone NPs (32).

#### 2.1.2 Starch

Starch is a naturally biocompatible and biodegradable polymer. There are two different types of starch: amylose and amylopectin. Most starch consists of ~20% amylose and ~80% amylopectin. While amylose and amylopectin are readily degraded by α-amylase, starch remains insoluble in water (33). Starch microparticles can be utilized as a vaccine carrier because it prevents the denature of antigen proteins by temporarily protecting antigens from the acidic and enzymatic environment of mucosal surfaces (34). Protein molecules can be encapsulated and coupled into particles made of modified and cross-linked starch. Starch can be shaped in a variety of forms, including Spherex microparticles, polyacryl starch microparticles, silicone-grafted starch microparticles, and more (33). Studies have shown that macrophages stimulated by starch in the form of granules release IL-1 (35). In the meantime, mice immunized intranasally with an antigen and silicone-polymer-grafted starch microparticle system likewise produced a robust circulating IgG responses (34, 36).

Pullulan, a microbial exopolysaccharide generated by *Aureobasidium pullulans*, is a highly biocompatible and biodegradable polymer that is odorless, tasteless, non-toxic and edible. It has maltotriose repeating units and is a linear, unbranched polymer chain structurally (37). A nanogel particle consisting of a cholesterol-bearing pullulan has been proposed as an intranasal delivery system (38). Charge-based interaction can be employed to make the particles stick to the nasal epithelium and release antigens since the particles have a positive charge while the mucosa has a negative charge (39). This mechanism can slow the release of antigens and prolong the existence time of antigens in the nasal mucosa (40). The antigen can be efficiently delivered to the nasal epithelium, where it is then taken up by dendritic cells. A protective immune response against *Streptococcus pneumoniae* was induced in mice after they were intranasally immunized with a vaccine conjugated with pneumococcal surface protein A using a cationic cholesteryl-group-bearing pullulan nanogel as a delivery system. This resulted in an increased production of serum PspA-specific IgG1 antibodies, high levels of mucosal antigen-specific sIgA antibodies, Th17 and Th2 responses, and the growth of bacteria in the mice’s lungs and nasal cavities was inhibited (41). Furthermore, the vaccine was administered to rhesus macaques, a non-human primate species. It not only induced systemic and mucosal antibody responses in the macaques, but it also prevented the antigen from being deposited in their brains or olfactory bulbs, making it safe (42). A recent study investigated the utilization of pullulan-bearing cholesterol moieties in nanogel particles as a delivery system. The delivery system induced antigen-specific serum IgG and nasal mucosal IgA by intranasally immunizing mice with pneumococcal surface protein A as an antigen (43).

#### 2.1.3 Maltodextrin

Maltodextrin (MD) is a natural polysaccharide derived from partially hydrolyzed starch. Maltodextrin-based nanoparticles, known as supramolecular biovectors (SMBVs) and cationic porous maltodextrin nanoparticles (NPLs), can be mixed with lipids to be employed as intranasal delivery systems (44).

After intranasal immunization, lipid-coated SMBVs with maltodextrin scaffolds cling to the mucosa continuously, enhancing intranasal antigen delivery and inducing systemic and mucosal immunological responses, such as antibody production and Th1 and Th2 immune responses (45). The efficacy of SMBV-based nasal influenza vaccinations was demonstrated in a dose-escalation clinical trial that was randomized, placebo-controlled, and conducted on healthy individuals. The vaccines produced mucosal immune responses and were well tolerated with moderate immunogenicity (46).

With anionic lipids contained in the maltodextrin scaffold as the core, Maltodextrin nanoparticles (MdNP) is a porous cationic maltodextrin scaffold and has been investigated as nasal route delivery methods for antigens and medications (47, 48). Regarding the safety of maltodextrin, numerous *in vivo* and *in vitro* tests have demonstrated that MdNP is safe since it is unable to penetrate the nasal mucosal epithelial barrier (48). When compared to other nanoparticle delivery systems, MdNP also had the longest residence duration in dendritic cells, macrophages, and airway epithelial cells, the highest endocytosis efficiency, and the strongest antigen delivery effect (49). The immune response induced by MdNP is dominated by Th1/Th17 (44). MdNP has been utilized to prevent acute or chronic toxoplasma gondii infection (50). According to a recent study, mice inoculated with the MdNP formulations were not only protected against infection, but also did not transmit the influenza virus (51).

#### 2.1.4 Alginate

Alginate is the basic carbohydrate component of brown algae. It is an unbranched copolymer with the qualities of hydrophilicity, adhesion, biocompatibility and biodegradability (52–54). Gel microparticles are formed when most bivalent cations are added to alginate solutions (52). The resulting microparticles are irregular in shape, have a rough and porous surface, and a high polypoid structure. These particles can develop under more hospitable settings (55). Their benefits include being inexpensive and non-toxic at the same time (52). Alginate has strong adhesion and can extend the duration of contact between microparticles and M cells, hence augmenting antigen absorption (55). By activating macrophage-like cells through the NF-κB pathway, alginate can trigger an innate immune response (56). Alginate microparticles have been employed in the creation of Klebsiella pneumoniae vaccines (55).

Alginate nanoparticles are often negatively charged, and mucus on the mucosal surface is similarly negatively charged, so this makes it unsuitable for substance delivery. Chitosan and alginate are frequently used in combination because the inclusion of chitosan enhances the positive charge on the particle surface while also enhancing the immunostimulatory qualities and stability of chitosan (54, 57). Chitosan/alginate nanoparticle encapsulated bee venom (BV), CH/AL-BV, has slow-releasing properties and mucosal adhesiveness. Nasal delivery of CH/AL-BV in pigs enhanced Th1-related responses, porcine reproductive and respiratory syndrome virus (PRRSV)-specific viral neutralizing antibody and significant reductions in PRRSV load in serum, lung and bronchial lymph nodes (54). Recently, nasal immunization of mice with trimethylchitosan nanoparticles containing influenza virus coated with sodium alginate induced a significant increase in the IgG2a/IgG1 ratio and improved immune protection against the virus (57). The combination of chitosan and alginate nanoparticles has the potential to be a highly effective vaccine delivery system.

Hyaluronic acid and dextran are other polysaccharides that can be also utilized as vaccine delivery platform. As a nanoparticle vaccine delivery system, a biodegradable polymer micellar coated with hyaluronic acid was created. Ovalbumin and adjuvant CpG-DNA were loaded onto the micellar, increasing the expression of MHC-II also IFN-γ and IL-4 mRNA expression in mouse dendritic cells. Correspondingly, nasal immunization resulted in higher antigen-specific serum IgG and nasal IgA levels (58).

### 2.2 PLGA: a promising and thriving mediators for delivery system

PLGA, poly (lactic-co-glycolic acid), is a biodegradable polymer consisting of lactic acid and glycolic acid linked via ester linkages. PLGA is soluble in common solvents such as acetone, chlorinated solvents, and ethyl acetate, and can be processed into virtually any shape and size, and encapsulated into various molecules (59). PLGA nanoparticles are one of the most widely used particles, having been used in a wide range of cosmetic, food, agricultural, and drug delivery systems (60).

Among vaccine delivery systems, PLGA is commonly used to protect peptide vaccines from degradation, enhance antigen absorption by APCs, and stimulate robust T cell immune responses (61). Furthermore, PLGA nanoparticle vaccines allow for a sustained release of antigen, which is essential for the duration and intensity of the induced vaccine response. Since PLGA nanoparticles are biodegradable, biocompatible, and non-toxic, they have been approved for many biomedical applications, with the U.S. FDA approving them as sustained-release drug delivery systems (62).

Büyükbayraktar et al. developed an anti-tuberculosis vaccine with polycation-coated PLGA nanoparticles as delivery system, a potential vaccine model for long-term pulsatile release of antigenic peptide of ESAT-6 protein of M. tuberculosis, which augment the immunostimulation and also allows to be administered via nasal route. Using *in vitro* model of murine macrophage cell line (J774) and human macrophage cell line (THP-1), the peptide-loaded nanoparticle enhanced the immunogenicity of the peptides to induce more nitric oxide (NO) than the free peptide and non-coated nanoparticle, demonstrating the immunostimulant activity of the nanoparticle systems. They also discovered that the macrophages exposure to these formulations maintained their intact structure without any cytotoxic effects (63).

Additionally, a number of studies using a model antigen such as tetanus or diphtheria confirmed the potent adjuvant effect of PLGA, which connected with their capacity to be effectively taken up by APCs (61). Many studies also tend to surface-modify it which can help them evade the body’s defense system, and make vaccines safer and more efficient through nasal immunization, a non-invasive route (59).

### 2.3 Gold nanoparticles (AuNPs) can capture and retain antigenic epitopes

Gold nanoparticles (AuNPs) represent one of the most widely studied metallic nanomaterials, which typically range in size from 1 nm to 100 nm, which may be beneficial in overcoming biological barriers (64). It is available in various types such as gold nanorods, nanocages, nanostars, nanocubes and nanospheres, and the type and size of the nanoparticles affect their physical characteristics and surface functionalization (65). Gold nanoparticles are widely used in the biomedical field due to their small size-to-volume ratio, functionalization, stability, low toxicity, and ease of detection, and they can bind to a variety of functionalized fractions through different types of interactions, including ligands, medicinal agents, DNA, amino acids, proteins, peptides, and oligonucleotides (66, 67).

Gold nanoparticles can capture and retain antigenic epitopes and improve the efficiency of antigen delivery and presentation. By studying different shapes of gold nanoparticles, Hongjuan Zhao et al. found that star-shaped AuNPs captured and retained more repetitive antigenic epitopes and triggered a strong humoral immune response mediated by the cooperation of CD4^+^ T helper cells and follicular B cells, whereas caged AuNPs caused a strong CD8^+^ T cell immunity (68).

Immunoreactivity of a DNA vaccine expressing the SARS-CoV-2 spike (S) protein on gold chitosan nanocarriers reveals a sustained surge in antibodies (IgG, IgA, and IgM) (69). In addition, coupling lipopolysaccharide (LPS) from Bacillus thailandensis with AuNPs for intranasal immunization of mice, which promote robust antigen-specific antibody responses (70).

### 2.4 Nanogels can retain the hydrated nature and shrink-swell properties of hydrogels

Nanogels are drug delivery vehicles with three-dimensional tunable porous structures with particle sizes in the submicron range, from 20 to 250 nm (71). It is formed by a system of chemically or physically cross-linked swellable polymer networks, which help encapsulate small molecules, oligonucleotides and even proteins while maintaining their structural integrity (72). As a hydrogel, nanogels can retain the highly hydrated nature and shrink-swell properties of hydrogels under different conditions, and these unique properties give nanogels the ability to enable drug delivery, diagnostics, and imaging (72, 73). Unlike typical nanoparticles, nanogels exhibit tunable particle size, particle shape, and sensitivity to pH, temperature, ionic strength, redox conditions, and other external stimuli. Nanogels have a pronounced spherical structure, and with the latest advances in their fabrication processes can also be produced in a variety of nanogel types, allowing for effective controlled drug release properties (74, 75).

Nanogel consisting of self-assembled cholesteryl group-bearing pullulan (CHP) has strong advantages as a novel adjuvant-free and safe carrier for mucosal vaccines. For example, nasal administration of nanogels containing subunit types of botulinum antigen (BoHc-nanogel) or pneumococcal surface protein A (PspA-nanogel) enhance antigen-specific systemic and mucosal immune responses, even in the absence of biologically active mucosal adjuvants (e.g., CT) (38).

Gel system consisting of glutamine and benzaldehyde derivatives combined with extracellular vesicles derived from mouse aortic endothelial cells has better absorption efficiency in the nasal cavity and reduces levels of pro-inflammatory Ly6C^hi^ monocytes/macrophages and neutrophils (76). Y Fukuyama et al. established a nanosized nasal vaccine delivery system by using a cationic cholesterol group-bearing pullulan nanogel (cCHP nanogel) in a study examining the central nervous system safety and efficacy of nasal vaccination with their developed cCHP nanogel containing pneumococcal surface protein A (PspA-nanogel) against pneumococcal infection in nonhuman primates, demonstrating that nasal PspA-nanogel vaccination is a safe and effective strategy for the development of a nasal vaccines for the prevention of pneumonia in humans (41).

### 2.5 Lipopeptides: self-assembly structure and efficient immune response

Lipopeptides are mixtures of lipids and peptides that have the ability to self-assemble into the structures of nanoparticles in an aqueous environment. The interaction or balance between the hydrophilic and lipophilic qualities of lipids and peptides determines the lipopeptides’ ability to self-assemble (77). Lipopeptides are used as delivery systems in nasal vaccines primarily to prevent rheumatic fever and rheumatic heart disease, which are significant respiratory infections caused by Group A streptococcus (GAS), which makes it an ideal delivery system, suitable for both mucosal and systemic immunization (78).

Because of their small nanosize structure and shape, lipopeptide vaccines are well absorbed by APCs and trigger a robust immune response and employed as a nasal vaccination to prevent GAS infection. For instance, the LP-88/30-J14 lipopeptide vaccine (containing both the N-terminal and C-terminal epitope of GAS) has a higher IgG titer and APC uptake of antigen due to its smaller nanosize, which increases the uptake of M protein antigen by DC, in comparison to LP-88/30 (N-terminal epitope) and LP-J14 (C-terminal epitope) alone (79).

### 2.6 Liposomes enhance the delivery of antigens to antigen-presenting cells

Lipid nanoparticles are submicron capsules with an aqueous core, such as liposomes, micelles, or nanoparticles with oil, solid, or a non-crystalline core surrounded and stabilized by a lipid layer, e.g., nucleic acid-containing lipid nanoparticles (commonly referred to as LNP) (80). Among them, liposomes are safe, biocompatible, biodegradable spherical nanoparticles composed of cholesterol and phospholipids that can be loaded with both hydrophilic and lipophilic molecules, and these properties make liposomes good gene, drug, and vaccine delivery systems (81). Liposomes can be cationic, anionic, or neutral, depending on the phospholipids that make up the liposome. Phospholipids are amphiphilic molecules with hydrophilic heads and lipophilic tails, and the surface charge of liposomes depends on the phospholipid head group (82). Depending on the nature of the loaded substance, liposomes can be loaded with drugs or antigens in two ways, either by encapsulating a water-soluble substance in a hydrophilic core or by containing a lipid-soluble substance in the hydrophobic space of a bilayer lipid membrane (82).

Liposomes enhance the delivery of antigens to antigen-presenting cells and co-deliver antigens with adjuvants, thereby increasing vaccine efficacy and enhancing immunogenicity (83). Cationic liposomes are widely used for gene and protein delivery. Cationic liposomes have been used in nasal immunization (84). Cationic DOTAP/DC-chol liposomal complex OVA drops preferentially induced the Th2 response after nasal immunization and enhanced OVA uptake by CD11c^+^ DCs in nasal-associated lymphoid tissues (85). In addition, it has also been shown that immunization with cationic liposomes complexed with antigen followed by nasal drops enhances antigen-specific mucosal IgA responses by inducing IL-6 expression. Intranasal liposomal vaccine delivery induces a mucosal response in the respiratory system and a systemic immune response that produces IgA and systemic IgG (86).

In addition, polymeric micelles are considered good carriers for drug delivery due to their stability, nanosize, surface properties, and enhanced permeability and retention effects (87). The hydrophobic core of the micelles consists mainly of polyester, poly (L-amino acids) and polycaprolactone, and the hydrophilic shell consists mainly of polyethylene glycol (PEG), with sizes ranging from 10 to 100 nanometers (88, 89). Polymeric micelle-based antigen delivery systems were developed for bacterial pathogens. Shaobin Shang et al. loaded mycolic acid (MA), a lipid components of Mycobacterium tuberculosis (Mtb) cell wall, into micellar nanocarriers (MA-Mc), which elicited a CD1b-restricted T cell response in the lungs after intranasal immunization of mice, demonstrating that MA-Mc can be explored as subunit vaccines against Mtb infection (90). Kengo Suzuki et al. found that hyaluronic acid (HA)-coated micelles can efficiently deliver antigens and adjuvants to mucosal resident immune cells, and that HA-coated micelles are a promising platform for the development of nasal vaccines against infectious diseases (58).

### 2.7 VLPs can be easily detected by immune system cells

Virus-like particles (VLPs) are multimeric self-assembled particles made up of one or more structural proteins that lack viral genetic material, making them incapable of infecting the host cell (91). As in the case of virus capsids, the antigenic proteins of VLPs self-assemble into highly symmetrical and strict architectures, usually icosahedral and octahedral, with the most of VLPs’ size ranging from 20-200nm (92). Virus-like particles can be produced using a variety of expression systems (ESs) without the requirement to propagate pathogenic viruses. The ESs include bacteria, yeast, insect cell lines, plants, mammalian cell lines and cell-free ESs (92). VLPs are used for a variety of purposes. Because they contain an internal cavity, they can be used as carriers for the delivery of bio- and nanomaterials, including drugs, vaccines, quantum dots and imaging substances (93). Using VLPs as a delivery strategy has numerous benefits, including precise targeting, biocompatibility, and biodegradability (94).

Vaccines based on virus-like particles have gained popularity due to their high immunogenicity. Because of their underlying geometry, VLPs resemble pathogen-associated structural patterns (PASP), which are easily detected by immune system cells (93). As a result, VLPs are excellent for cellular phagocytosis and antigen presentation of dendritic cells (DCs), eliciting both robust cellular and humoral immune responses even without an adjuvant (91, 95).

To date, numerous perilous viral pathogens have been mimicked by VLPs, including Severe Acute Respiratory Syndrome Coronavirus 2 (SARS-CoV-2); Hand, foot and mouth disease virus. Authorized VLP vaccines against hepatitis B virus (HBV), hepatitis E virus (HEV), and human papillomavirus (HPV) are globally used and many others are in clinical trials (92, 96). VLPs are readily absorbed by APCs, particularly DCs, which improves interactions with NALT. Furthermore, VLP-based intranasal vaccinations can efficiently boost mucosal immune responses, hence reducing viral shedding and local transmission (97). These benefits have made VLP-based vaccinations popular in the development of intranasal vaccines. Rothen et al. described a COVID-19 vaccine based on virus-like particles (VLPs) for intranasal administration. Intranasal delivery of the CuMV_TT_-RBD vaccine induces robust local and systemic immune responses, generating high-avidity antibodies with broad-spectrum neutralization against SARS-CoV-2 and its variants of concern (VOCs) in a murine model. This work establishes a foundational framework for advancing next-generation mucosal COVID-19 vaccines designed to block viral entry at respiratory portals while curbing transmission (98).

## 3 Nanoemulsion enhances adhesion and immune response

Nanoemulsion (NE) is composed of water in oil (W/O) or oil in water (O/W), surfactants and cosurfactants, with an average droplet diameter of less than 500nm. Long-chain triglycerides (LCT) and medium-chain triglycerides (MCT) are the two categories of commonly utilized oils (99). Ionic and non-ionic surfactants are the two categories of commonly used surfactants. Non-ionic surfactants include Tween, Span, Poloxamer, etc., while ionic surfactants include Cetylpyridinium Chloride (CPC), Benzalkonium Chloride (BCI), etc (100).

Nanoemulsion with good adhesion enhances the duration of antigen residency in the nasal cavity, hence increasing the capacity of APC to absorb antigens and potently stimulating the immune system. The physical and chemical characteristics of surfactants may be connected to the robust NE adherence to mucosa. The cationic surfactant CPC interacts with the negatively charged mucin and the non-ionic surfactant Tween interacts with the mucin due to its hydrophilicity, both of which can make NE firmly adhere to the surface of nasal mucosa, promote the uptake of antigen by nasal epithelial cells, and thus enhance the specific humoral immune response (100).

NE such as MF59 and W_80_5EC are used for intranasal immunization. MF59, an oil-in-water emulsion (O/W), is primarily used for influenza vaccine (101). A W_80_5EC-based influenza vaccine has been successfully tested in a Phase 1 randomized, controlled, observer blind clinical trial, showing a good safety profile in healthy adult volunteers, and induced systemic and mucosal immunity after a single intranasal vaccination (102). Furthermore, in mice, rats, and guinea pigs, the NE-based hepatitis B vaccine has demonstrated significant immunogenicity and good safety (103). According to research on vaccines against Helicobacter pylori infection, NE vaccine has high delivery efficiency and no obvious cytotoxicity, induces effective specific Th1 response, reduces the colonization of Helicobacter pylori (104). One study has developed an adjuvant integrating NE that activates TLR2/4 and NLRP3 with an RNA agonist of RIG-I (IVTDI). The spike protein vaccine incorporating NE/IVD significantly enhances a Th1-biased cellular immune response and elicits high neutralizing antibody titers (105).

Recently, a new nano-adjuvant, O/ILNE, consisting of an ionic liquid of choline (+) and niacin (–) instead of water. In contrast to MF59 emulsion (O/W), O/IL NE has been shown to increase mucosal IgA, systemic IgG, and cellular immunity. Therefore, the prolonged retention of antigens in the nasal cavity and improved paracellular transport of antigens to the submucosa make it an incredibly effective delivery strategy (106).

## 4 Probiotics in intranasal delivery system

The WHO defines probiotics as living microorganisms which administered in adequate amounts confer a health benefit on the host (107). Good safety, adherence, and availability make probiotics highly promising for use in vaccine administration (108, 109).

### 4.1 Bacterial spores: a stable and effective mucosal vaccine delivery system

Bacillus spores can be used as mucosal vaccine carriers (110). Among them, *Bacillus subtilis* spores have the advantages of thermal stability, and non-pathogenicity and can be used as a vaccine delivery system (111). *Bacillus subtilis* is a Gram-positive non-pathogenic and endospore-forming bacterium (112). *Bacillus subtilis* spores can sprout, develop, and revert to mature bacteria when the surrounding conditions are right (113). The resistance and stability of the spore are determined by its unique structure (110). Chromosome copies are found in the spore’s core, which is encased and shielded by a protein shell and peptidoglycan (110). Isticato R et al. reported that mutant spores with modified shells are better at absorbing antigens than spores of the wild type (114). *Bacillus subtilis* spores can activate innate immunity via TLRs and promote DC maturation and NK cell recruitment. Intranasal immunization of spores absorbed with antigen increased the uptake of antigen, raises cytokine levels and sIgA levels, and conferred protection against viral challenge (114, 115).

Recently, a study using *Bacillus subtilis* spores coupled with Plasmodium falciparum recombinant protein to intranasally immunize mice showed that the spores increased the immunogenicity of the antigen and induced higher levels of serum IgG (116). Studies have also been conducted to create vaccines against C. difficile (Clostridioides difficile) spores by adsorbing C. difficile spore surface proteins to *Bacillus subtilis* spores (117, 118). Furthermore, studies that adsorbed tetanus toxin to *Bacillus subtilis* spores and immunized mice intranasally showed that spore treatment increased tetanus toxin-specific sIgA production and resulted in more rapid serum IgG production (119).

### 4.2 Others

Apart from *Bacillus subtilis* spores, mucosal vaccines can also be delivered intranasally via Escherichia coli and Lactic acid bacteria. Some studies have constructed *Escherichia coli* expressing allergens and demonstrated that intranasal pretreatment with this *Escherichia coli* before multiple sensitization in mice can significantly reduce allergen-specific serum IgE levels and lung inflammation (120). Lactic acid bacteria are a group of Gram-positive, non-sporogenic bacteria, including Lactobacillus, Lactococcus, etc (121). Pneumococcus and tetanus vaccinations have been developed using lactic acid bacteria as a delivery system to stimulate a favorable immune response (122, 123).

## 5 Limitations and safety concerns of intranasal vaccine delivery system

While intranasal vaccines offer significant advantages, their development and application are constrained by anatomical, physiological, and technical challenges.

### 5.1 Anatomical and physiological risks

The nasal cavity’s proximity to the central nervous system poses unique risks. The thin olfactory epithelium-blood-brain barrier raises concerns about neurotoxic effects if vaccine components inadvertently enter the brain. For instance, mild adverse effects such as rhinorrhea and nasal congestion are common, while severe complications—though rare—have been documented (12, 124, 125). A notable example is the NasalFlu vaccine (approved in Switzerland in 2001), which was withdrawn due to reports of Bell’s palsy in immunized individuals (126).

### 5.2 Technical limitations of delivery systems

Current delivery platforms face three major hurdles: (i) Inefficient Antigen Release: Many carriers, such as PLGA nanoparticles, exhibit incomplete antigen release and cannot undergo sterile filtration, limiting their clinical scalability (127). (ii) Manufacturing Complexity: Materials like chitosan require chemical modification to improve water solubility and biocompatibility, adding to production costs (128). (iii) Carrier-Related Toxicity: Certain formulations, including cationic liposomes, may induce dose-dependent inflammation, genotoxicity, or cell membrane disruption (129, 130).

### 5.3 Safety evidence from preclinical studies

Emerging preclinical data highlight potential safety issues: (i) Chitosan-based nanoparticles have shown dose-dependent developmental toxicity in zebrafish embryos, including reduced hatching rates, increased malformations, and neurobehavioral abnormalities (131). (ii) Acylated starch nanoparticles exhibit moderate cytotoxicity at high concentrations, though low doses remain tolerable (132). (iii) PLGA particles may trigger local immune overactivation if antigen release kinetics are poorly controlled (127). Despite these challenges, systematic safety evaluations remain limited, underscoring the need for standardized toxicity assays and long-term monitoring in future studies.

## 6 Conclusions

**Figure 3:**
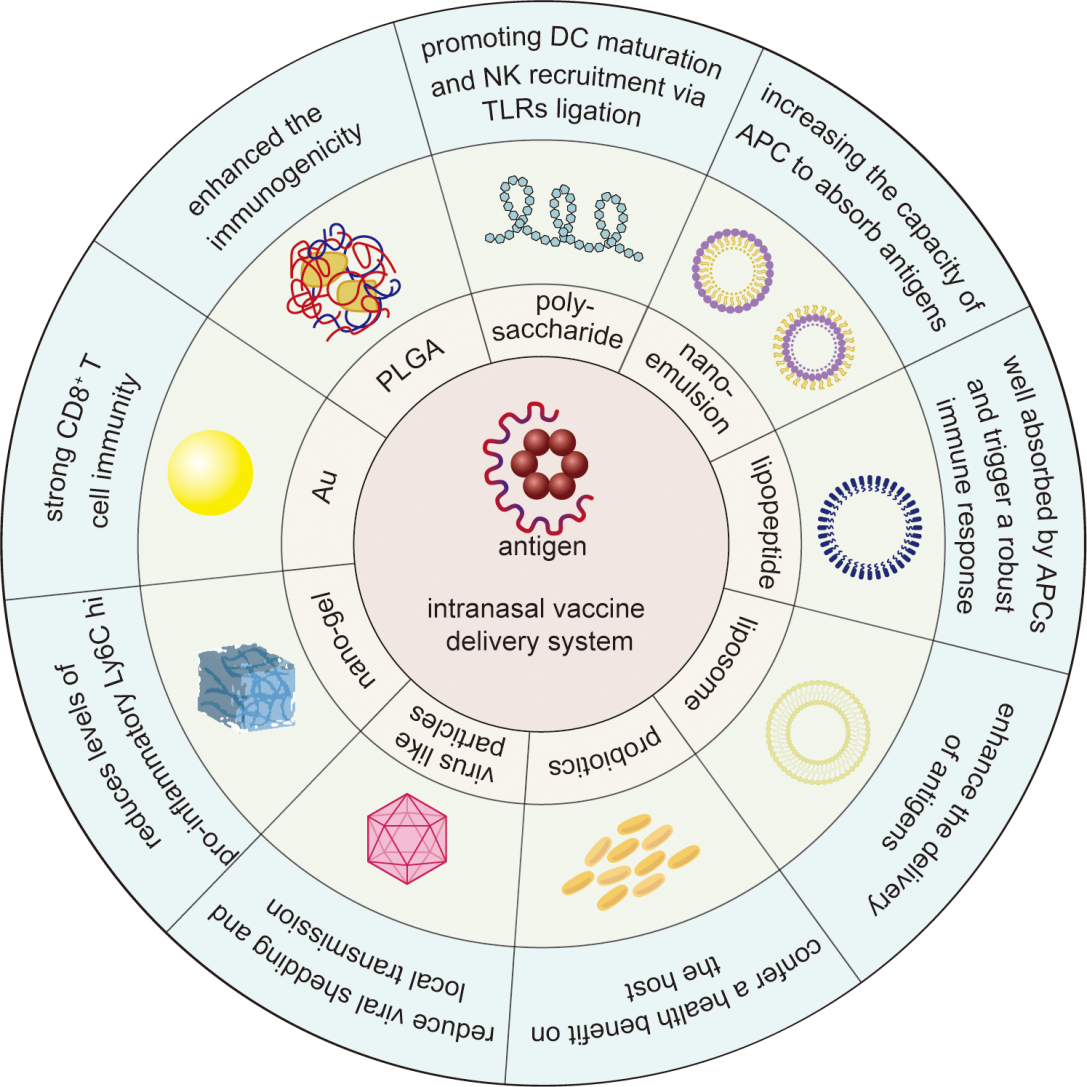
Schematic overview of intranasal vaccine delivery system against infectious disease. The outer blue segment and the green segment in the middle represents the key outcome induced by and the diagram of intranasal delivery system, respectively. Polysaccharides stimulate dendritic cell maturation and natural killer cell recruitment through TLR ligation. Nanoemulsions enhance antigen uptake in antigen-presenting cells. Lipopeptides boost APC absorption and activate potent immune responses. Liposomes optimize antigen delivery efficiency. Probiotics strengthen host health. Virus-like particles inhibit viral shedding and curb local transmission. Nanogels suppress pro-inflammatory Ly6C hi levels. Au nanoparticles elicit robust CD8^+^ T cell immunity. PLGA nanoparticles amplify antigen immunogenicity.

Intranasal immunization is a safe, effective, and convenient route of mucosal immunization. The nasal cavity itself, with its enormous surface area and specialized immunological tissue (NALT), is an efficient entry channel for vaccinations, making intranasal immunization promising for the mucosal routes. However, intranasal cilia clearance, mucus barrier, and enzyme milieu which make intranasal delivery of antigens challenging. Various intranasal delivery technologies, such as nanoparticles and nanoemulsions, have been created to improve vaccine distribution, increase antigen penetration, and elicit more robust immune responses to solve these problems (**Figure 3**). Among diverse types of delivery system, nanoparticles especially chitosan nanoparticles are well-studied and offer a promising delivery strategy for promoting antigen uptake and lowering unfavorable vaccine reactions. Despite the promising results in animal models, clinical findings were limited. Therefore, further studies are needed to evaluate the efficiency and safety of these delivery system in human studies. With advances in delivery technologies such as nanoparticles, the challenges of antigen degradation and malabsorption can be mitigated, which may lead to more efficient and broader vaccination strategies. Furthermore, combining advanced delivery systems with adjuvants including STING-activating (e.g., CDNs) could represent a powerful strategy to amplify mucosal immunity. Co-delivery of antigens and adjuvants within nanocarriers may ensure spatiotemporal synchronization of antigen presentation and innate immune activation, thereby enhancing vaccine efficacy. These advances have the potential to increase vaccine efficacy and acceptance, particularly in the prevention of respiratory infections such as influenza, respiratory syncytial virus and coronavirus, and will play an increasingly critical role in global vaccination campaigns and contribute to improved global public health outcomes.

## Glossary

APCs: antigen presenting cells
AuNPs: Gold nanoparticles
BALF: bronchoalveolar lavage fluid
Bm: Burkholderia mallei
BoHc: botulinum type A neurotoxin
BV: Bee venom
cCHP: Cationic-type CHP
Chi-C48/80: compound 48/80 loaded chitosan
Chi-PCL: Chitosan-poly epsilon caprolactone
CHP: cholesteryl group-bearing pullulan
CH/AL-BV: chitosan/alginate nanoparticle encapsulated bee venom
C-O-NP: curdlan-chitosan conjugate nanoparticle
CPC: Cetylpyridinium Chloride
CS: curdlan sulfate
CTA1-DD: cholera toxin A1 subunit -dimer of the D-fragment of protein A from Staphylococcus aureus bacteria
CT: cholera toxin
CTB: cholera toxin B subunit
DGNP: Dendritic Gold Nanoparticles
DOTAP: 1,2-dioleoyl-3-trimethylammonium-propane
DT: diphtheria toxoid
ESs: expression systems
ESAT: Early Secretory Antigenic Target
GAS: Group A Streptococcus
GC: glycol chitosan
HA: hyaluronic acid
HAS: Human serum albumin
HBsAg: hepatitis B surface antigen
HBV: hepatitis B virus
HpaA: Helicobacter pylori adhesin A
HPV: human papillomavirus
IFN-I: type I interferons
IFN-γ: interferon-gamma
IgA: immunoglobulin A
IgG: immunoglobulin G
IL-1: interleukin-1
IL-6: Interleukin-6
IVT DI: an RNA agonist of RIG-I
LNP: Lipid Nano Particle
LP: Lipopeptides
LPS: lipopolysaccharide
MA: mycolic acid
MA-Mc: MA-loaded micellar
MD: Maltodextrin
MdNP: Maltodextrin nanoparticles
Mtb: Mycobacterium tuberculosis
NALT: nasal-associated lymphoid tissue
NE: nanoemulsion
NF-κB: nuclear factor kappa-B
NPL: nanoparticles lipid
O-HTCC: O-(2-hydroxyl) propyl-3-trimethyl ammonium chitosan chloride
OVA: ovalbumin
PASP: pathogen-associated structural patterns
PEG: polyethylene glycol
PLGA: poly (lactic-co-glycolic acid)
PRRSV: porcine reproductive and respiratory syndrome virus
PspA: pneumococcal surface protein A
PVP: poly (4-vinylpyridine)
rPfCSP: recombinant P. falciparum circumsporozoite surface protein
SARS-CoV-2: Severe Acute Respiratory Syndrome Coronavirus 2
SMBVs: supramolecular biovectors
S-RBD: spike receptor binding domain
TE: total extract of Toxoplasma gondii antigens
Th1: type-1 helper T cell
Th2: type-2 helper T cell
Th17: type-17 helper T cell
TS-PDMS-grafted MP: 3-(triethoxysilyl)-propyl-terminated polydimethylsiloxane-grafted microparticles
TTFC: C fragment of the tetanus toxin
VOCs: variants of concern
